# The impact of external environment on pulmonary development – a morphological evaluation of pulmonary tissue in preterm infants

**DOI:** 10.25122/jml-2025-0045

**Published:** 2025-04

**Authors:** Raluca Chirculescu, Paul Cristian Balanescu, Gheorghe Peltecu

**Affiliations:** 1 Carol Davila University of Medicine and Pharmacy, Bucharest, Romania; 2 Department of Pathology, Filantropia Clinical Hospital, Bucharest, Romania; 3 Department of Internal Medicine, Colentina Clinical Hospital, Bucharest, Romania; 4 Department of Obstetrics and Gynecology, Ponderas Academic Hospital, Bucharest, Romania

**Keywords:** preterm birth, pulmonary morphology, lung development, branching morphogenesis, alveoli

## Abstract

Preterm birth disrupts the natural progression of pulmonary development, which can trigger functional and morphological consequences that may lead to death or the development of a chronic lung disease. The objective of this research was to evaluate the pulmonary morphological characteristics in 67 preterm neonates who had survived for a minimum of 24 hours. All evaluations were carried out on paraffin-embedded lung tissue, sliced at 5 micrometers, and stained with a standard hematoxylin-eosin stain. From each case, photomicrographs of one square millimeter were assessed, and the quantity of alveoli, the diameter of the alveoli, the thickness of the alveolar septum, and the total thickness of the arteriolar and venular walls were measured. The research findings revealed that prolonged oxygen therapy has an impact on the density of alveoli per square millimeter in premature infants, regardless of their gestational age at birth. Additionally, neonates with lobar lung abnormalities exhibit a reduced number of alveoli per square millimeter. Moreover, preterm neonates delivered at extreme gestational ages demonstrated a notably reduced alveolar diameter compared to those born at more advanced gestational ages, and infants who developed bronchopulmonary dysplasia may exhibit increased alveolar septal thickness compared to other newborns.

## INTRODUCTION

Preterm birth persists as a major public health concern, serving as a significant contributor to neonatal morbidity and mortality. Pulmonary development commences in the early stages, during the embryonic period, progresses throughout the fetal stage, and culminates postnatally in early adulthood [1-3].

The embryonic period, encompassing the initial 8 weeks of gestation is responsible for organogenesis, a crucial process of tissue growth and differentiation in organs. Originating from the endoderm, the respiratory system is fully formed and ready for maturation by the end of the 8 week of gestation. After 9 weeks of gestation, the fetal period begins, and the respiratory system undergoes four stages of maturation (pseudo-glandular, canalicular, saccular, and alveolar) [2,3]. Each stage of lung development is characterized by distinctive morphological changes that synergistically contribute to the formation of a structurally and functionally intricate organ [1]. However, the precise temporal boundaries of fetal lung development remain ambiguous and may exhibit overlapping characteristics [4-6]. The survival of preterm newborns becomes possible with the formation of gas exchange membranes, which corresponds to the end of the canalicular stage of lung development [3]. The adaptation of the newborn to extrauterine life is dependent on the acquisition of structural and functional pulmonary maturity. Postnatal lung development encompasses two important elements: the alveolar phase and the maturation of pulmonary microvascularization [1]. It is estimated that a term newborn has a gas exchange area of nearly 4–5 square meters and an average of 50 million alveoli [5]. The intricate process of alveolarization and microvascular maturation persists postnatally until early adulthood, although at a diminished rate compared to intrauterine life [7]. In the first 2 years following birth, there is a rapid increase in the number of alveoli, resulting in a gas exchange surface area of up to 100 square meters and a total of approximately 480 million alveoli [5,8]. Postnatal pulmonary development throughout adulthood is sustained by experimental investigations conducted in various animal species, revealing compensatory lung expansion subsequent to pneumonectomy [9,10]. The maturation of pulmonary microvascularization is an ongoing phenomenon that persists as long as alveolar structures are still being formed [7].

Starting from all these data in the literature and having an understanding of the sequence of lung development, a pertinent question emerges: What happens to lung development when it is disrupted by preterm birth? Will it be able to proceed along its natural development? How might external factors impact subsequent pulmonary maturation?

Morphometric measurements of human lung tissue proved challenging to assess and interpret on paraffin-embedded lung tissue, primarily due to the limited availability of lung tissue samples for evaluation. For this reason, morphometric assessments primarily rely on estimates derived from various modern techniques.

This study does not aim to provide a morphometric evaluation of the pulmonary tissue examination but rather to assess the morphological characteristics of the pulmonary tissue following exposure to the extrauterine environment and neonatal therapeutic interventions.

## MATERIAL AND METHODS

### Population study

#### Case selection

The retrospective observational study was conducted in the Department of Pathology at two medical centers: The National Institute for Mother and Child Health 'Alessandrescu-Rusescu' in Bucharest, Romania, and Filantropia Clinical Hospital in Bucharest, Romania, from January 2017 to December 2024. Maternal and neonatal medical data were obtained from medical and postmortem reports.

#### Inclusion criteria

Newborns who met the following criteria were eligible for inclusion in the study:

- Gestational age at birth less than 37 weeks.
- Minimum lifespan of at least 24 hours.

#### Exclusion criteria

Newborns who met any of the following criteria were excluded from the study:

- Term newborns.
- Lifespan less than 24 hours.
- Congenital pulmonary hypoplasia.
- Congenital diaphragmatic hernia.

### Sample size, data collection, and morphological studies

After identifying the eligible cases from both centers, a cohort of 67 autopsies was selected. We reassessed maternal and neonatal medical records, gathering information on obstetric history, prenatal clinical details, lifespan, duration of oxygen therapy, other treatments, and the occurrence of lobar lung anomalies.

Following the initial processing of the lung tissue samples (paraffin-embedded lung tissue - donor block) from each individual, small samples were collected, and a tissue microarray was created. Investigators assigned a distinct registration code to each case to prevent identification.

The tissue microarray was sectioned at a thickness of 5 micrometers and stained with standard hematoxylin-eosin stain, followed by evaluation under the optical microscope.

### Digital image analysis

From each case, microphotographs of one square millimeter were captured utilizing the Nikon Eclipse Si trinocular microscope at 200x magnification and the M-Shot digital camera MSX2 with 12.5 megapixels. For the evaluation and measurement of microscopic images, we utilized the Mshot Digital Imaging Software Version 1.1.6.

With the assistance of this program, each case was assessed for the number of alveoli per square millimeter, the maximum and minimum thickness of the alveolar septum, the maximum and minimum diameter of the alveoli, and the thickness of the arteriolar and venular walls.

To determine the density of alveoli per square millimeter, the aerial spaces were meticulously counted, and these aerial spaces were described as optical voids encircled by alveolar septa. It is important to note that any bronchial structures were intentionally omitted from this examination.

To establish the maximum and minimum diameter of the alveoli, a horizontal line was drawn on the microscopic image, connecting two opposing alveolar septa. A similar technique was used to assess the thickness of the alveolar septa and vascular walls.

### Statistical analysis and data interpretation

Statistical analysis was conducted utilizing the IBM SPSS software, version 28. Continuous variables exhibiting normal distribution were presented as mean values accompanied by standard deviation, whereas nominal variables were expressed in terms of frequencies and percentages. The differences between the two groups were examined using the unpaired Student’s *t*-test. The significance level was set at *P* < 0.05, and the Pearson correlation coefficient was utilized to assess the statistical relationship and connection between the data.

## RESULTS

**Figure 1:**
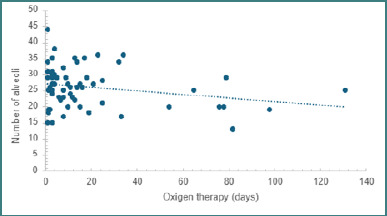
Prolonged effects of oxygen therapy on the number of alveoli

The 67 eligible postmortem examinations were selected from the Pathology Department of the National Institute for Mother and Child Health 'Alessandrescu-Rusescu' Bucharest between 2018-2020 and Filantropia Clinical Hospital Bucharest between 2017-2024. Among these, 37 were male and 30 were female, with 62.7% being delivered via cesarean section. The mean gestational age at birth was 28.9 +/- 3.13 weeks, spanning from 23 to 35 weeks gestational age, with an average lifespan of 21.6 +/- 33.45 days, ranging from a minimum of 1 day to a maximum of 149 days. In the observed cohort, each newborn received oxygen therapy for an average duration of 17.9 +/- 26.52 days, extending from a minimum of 1 day to a maximum of 131 days. The duration of oxygen therapy depended on the individual's lifespan. Due to the prolonged oxygen therapy, the impact of this treatment on the morphological characteristics of lung tissue was investigated. The findings revealed a substantial correlation between oxygen therapy duration and alveolar density per square millimeter (*P* < 0.001; r = 0.92). Specifically, a longer period of oxygen therapy was associated with reduced alveolar density per square millimeter (Figure 1).

Another interesting observation was made within the cohort of neonates with lobar pulmonary abnormalities. Out of the total 67 newborns, 17.91% exhibited abnormalities in lung lobation; these infants demonstrated a significantly reduced number of alveoli per square millimeter compared to those without these anomalies (mean difference, -4.60; 95% CI, -7.56 to -1.65; *P* = 0.003) (Table 1).

**Figure 2:**
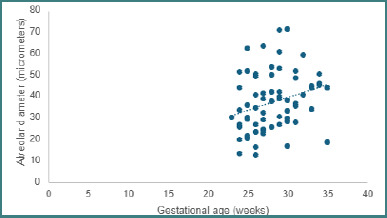
Minimum alveolar diameter in relation to gestational age at birth. The graph illustrates a negative correlation between gestational age and the density of alveoli per square millimeter, indicating that as the age of gestation decreases, the alveolar density also decreases.

When we evaluated the maximum and minimum diameters of the alveoli, we discovered that neonates with extreme prematurity (less than 28 weeks gestational age at birth) exhibited a notably diminished alveolar diameter (*P* = 0.006) in comparison to their counterparts born at more advanced gestational ages (Figure 2).

**Table 1:** A comparative analysis of the mean number of alveoli per square millimeter between the group presenting with lobar lung abnormalities and the group without lobar lung abnormalities

Criteria	Lobar lung abnormalities	No lobar lung abnormalities
Subject number	*n* = 12	*n* = 41
Mean	21.92	26.53
SD	± 3.423	± 6.775

*n*: the number of subjects expressed in absolute values (of the 67 neonates included in the study, 14 reports lacked any pertinent information related to pulmonary lobation abnormalities. The number indicated in the table refers to the 53 remaining neonates). SD: standard deviation.

**Figure 3:**
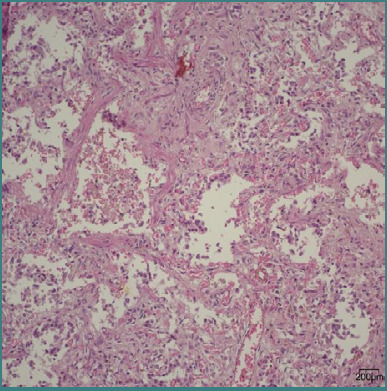
Microscopic image stained with standard hematoxylin-eosin stain, 200x magnification, revealing significant pulmonary fibrosis in a 29-week neonate who lived for 149 days.

**Figure 4:**
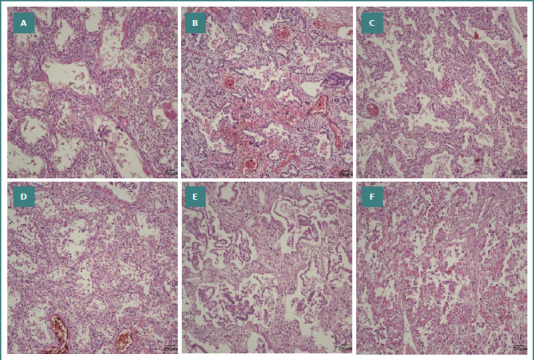
Microscopic images stained with standard hematoxylin-eosin stain, 200x magnification. A, pulmonary tissue from a 24-week neonate with a lifespan of 5 days; B, pulmonary tissue from a 26-week neonate with a lifespan of 6 days; C, pulmonary tissue from a 27-week neonate with a lifespan of 15 days; D, pulmonary tissue from a 29-week neonate with a lifespan of 9 days; E, pulmonary tissue from a 26-week neonate with a lifespan of 45 days; F, pulmonary tissue from a 30-week neonate with a lifespan of 104 days. The microscopic images show a prominent, thick alveolar septum with a rich mesenchymal matrix and a limited number of alveoli.

Of the total cases examined in the study, six neonates were born at a gestational age of less than 28 weeks, requiring an extended duration of oxygen therapy exceeding 28 days. These infants displayed a significant increase in the thickness of the alveolar septum (*P* = 0.019) (Figures 3 and 4 A-F).

Although other potential correlations have been explored, such as the impact of lifespan on the quantity of alveoli per square millimeter (*P* = 0.22) and the presence of alveoli per square millimeter in the cohort of children born to mothers with premature rupture of membranes or cerclage and pessary, no significant associations were observed between these variables (*P* = 0.67; *P* = 0.52). Also, in neonates with intrauterine growth restriction or those born to mothers who received antenatal treatment with corticosteroids, we did not observe a significant correlation between these parameters and the number of alveoli per square millimeter (*P* = 0.60; *P* = 0.77).

## DISCUSSION

To improve our understanding of the morphopathological changes discernible in the lungs of these preterm newborns, it is essential to comprehend the fundamental characteristics associated with each stage of fetal lung maturation. Based on morphological criteria, the fetal period of pulmonary development is segmented into four distinct stages.

The pseudoglandular stage is the first stage of the fetal period and begins soon after the embryonic period (weeks 8 to 16) [5]. The main purpose of this period is the formation of the bronchial tree, with branching morphogenesis leading to the formation of the first 20 generations of branching. During this period, the differentiation of the respiratory epithelium begins, forming the cilia of the columnar epithelial cells in the proximal airways. At the end of this period, in addition to terminal bronchioles, the arterial system, cartilage, and smooth muscle are also formed [1,2,5]. At this stage of lung development, gas exchange is not possible, and a fetus born during this period cannot survive.

In the canalicular stage (weeks 16 to 26), the bronchial branching continues, the pulmonary mesenchyme begins to decrease, and the capillary network comes into contact with the epithelial airspaces, forming the respiratory units [3]. The most important aspect of this period is undeniably the formation of the respiratory units, as it signifies the crucial transition to extrauterine existence, although lung development remains insufficient to sustain an autonomous life [2]. Another important event in lung development emerges at the 20^th^ week of gestation, marked by the appearance of lamellar bodies—intracellular structures responsible for surfactant storage—situated within the cytoplasm of type II pneumocytes [5]. Type I and II pneumocytes originate from bronchial epithelial cells. Following the emergence of type II pneumocytes, they actively contribute to the subsequent generation of type I pneumocytes, playing a crucial role in facilitating gas exchange [6,7]. Even though the survival of these preterm newborns becomes possible after the formation of the respiratory units and surfactant production, some may succumb due to inadequate gas exchange surface area and insufficient surfactant synthesis. The surviving fraction will necessitate prolonged intensive care, leading to substantial economic consequences [2].

The saccular stage (weeks 24 to 36) is characterized by the expansion of the gas exchange area through the secondary septation process, which involves the formation of new septa within the airspaces. This process results in an increase in the number of airspaces due to the formation of secondary septa originating from the primary septa. These secondary septa give rise to secondary crests housing a double-walled capillary network [7,11]. The lung mesenchyme undergoes significant reduction while the number of capillaries continues to rise, leading to the development of a sophisticated capillary network that facilitates efficient gas exchange. Consequently, the airspaces decrease in size, and the septa become thinner [5]. During this stage, branching morphogenesis ceases to be a feature [3,6]. Type I pneumocytes undergo a flattening process and contribute to the expansion of the alveolar-capillary barrier, whereas type II pneumocytes proliferate in both number and size, accumulating a greater quantity of surfactant [5]. Preterm neonates, born during this phase of pulmonary development, may face a challenging transition to extrauterine life due to deficiencies in gas exchange, surface area, and surfactant production. These inadequacies are exacerbated by the underdeveloped mesenchymal matrix and pulmonary vascularization [12].

The alveolar stage (week 36 to birth) - preceding birth, the alveolar sacs commence the formation of alveolar structures. The process of alveolarization initiates during intrauterine life and extends into the postpartum period [6]. To facilitate a more efficient and rapid gas exchange, the dual capillary network will transition into a singular network that connects two airspaces [2]. The alveolocapillary barrier must become as thin as possible, and the gaseous exchange area as large as possible. These two conditions are achieved during late lung development with the onset of alveolarization [11]. Throughout time, debates have arisen not only regarding the commencement of the alveolar phase but also its culmination. Some researchers have challenged the traditional beliefs regarding the development of alveoli prenatally. Their studies have demonstrated that alveolar structures can be detected in a diminished quantity starting from 32 weeks of gestation and that by 36 weeks of gestation, alveoli are uniformly distributed [5]. Nowadays, as a result of numerous studies conducted on laboratory animals and humans, it is widely recognized that the genesis of new septa and alveoli can occur throughout adulthood [4].

It has been reiterated several times in this article that preterm birth disrupts normal lung development, which is a complex process of interconnected events and mechanisms [13]. Lung development reaches a maximum point of functional development around ages 20-25, and then lung function begins to decline. Due to this long period of structural and functional pulmonary maturation, lung development can be influenced by various factors, including small gestational age at birth, as well as genetic and environmental factors [14]. Among the environmental factors that can impact lung function (maternal smoking, toxin exposure, alcohol intake), it is crucial to recognize that not only external elements play a role but also internal factors such as oxygen toxicity or infections [1,11,14]. Disruption of pulmonary development due to premature birth, particularly at a gestational age below 28 weeks, may lead to structural morphological alterations accompanied by varying degrees of impaired respiratory function [1,11,13]. Some studies suggest that the disruption of the process of lung development substantially increases the risk of developing chronic lung diseases such as asthma, chronic obstructive pulmonary disease (COPD), and interstitial lung diseases later in life [1].

A potential and relatively frequent cause of preterm birth is acute chorioamnionitis, resulting in premature rupture of membranes [13,15]. Infection during pregnancy is linked to an increase in surfactant production and a decrease in elastin production at the secondary crests, which are crucial in secondary septum formation [13]. The research did not find a significant correlation between maternal infection and the analyzed pulmonary morphological parameters. The correlation between chorioamnionitis and surfactant production is currently the subject of another ongoing study.

Knowing that the administration of antenatal corticosteroids stimulates lung maturation by accelerating the alveolarization process and enhancing surfactant production [16,17], we investigated whether there exists a correlation between this administration and the quantity of alveoli per square millimeter. The results obtained from our study group were inconsistent with the existing literature data. This might be attributed to the insufficient data available in the medical records to confirm whether all the cases we identified as having received antenatal corticosteroid treatment also adhered to the guidelines' recommendations. Moreover, the research was conducted using small lung tissue fragments, implying that the tissue samples may not have been sufficiently extensive to yield statistical significance. Additionally, as highlighted in the descriptive statistics findings, these neonates underwent an extended period of oxygen therapy (17.9 +/- 26.52 days), which had an impact on the alveolar count. A notable correlation was observed between the extended oxygen therapy and the alveolar count.

Anatomically, the right lung has three lobes delineated by two fissures, whereas the left lung consists of two lobes separated by a single fissure [18,19]. Of the neonates included in the research, 17.91% exhibited lobar pulmonary anomalies, predominantly affecting the right lung due to the absence of the oblique fissure. These pulmonary lobar anomalies denote aberrations in lung development, and our research has unveiled a statistically significant correlation between their presence and the low alveolar density per square millimeter compared to those without these anomalies.

Preterm neonates with extreme prematurity (below 28 weeks gestational age) exhibited a markedly diminished alveolar diameter in comparison to other newborns (*P* = 0.006). This observation can be explained by their birth during the canalicular stage, preceding the appearance of secondary crests that ultimately give rise to the secondary alveolar septum. In this phase of pulmonary development, the lung mesenchyme, although it begins to diminish, still retains its thickness, and the synthesis of elastin, crucial for pulmonary compliance, has not yet commenced.

Last but certainly not least, pulmonary fibrosis stands out as one of the most feared complications encountered in preterm infants who will develop chronic lung disease. In the past, this was the classic presentation of bronchopulmonary dysplasia, described over five decades ago by Northway [20,21]. Today, due to antenatal corticosteroid treatments and perinatal surfactant therapies, coupled with the implementation of advanced mechanical ventilation strategies, the incidence of pulmonary fibrosis has shown a significant decline, being replaced by the phenomenon of alveolar simplification [22]. In our research cohort, microscopic examination revealed pulmonary fibrosis in 10.44% of cases (*n* = 7), with six of these cases involving infants born at gestational ages below 28 weeks. These neonates displayed a significant increase in the thickness of the alveolar septum (*P* = 0.019), indicating that while the occurrence of pulmonary fibrosis has diminished over time, it still manifests in certain instances of extreme prematurity.

### Limitations

Although the region of pulmonary tissue underwent meticulous selection for analysis, the primary limitation of this study lies in the small sample size of lung tissue, measuring only one square millimeter. It is challenging to accurately capture the intricate alveolar, bronchial, and vascular structures in a microscopic image in a manner that accurately represents the entire lung tissue.

## CONCLUSION

Lung development is a dynamic process that continues for many years after birth. The disruption of the pulmonary development process leads to morphological and functional implications, escalating in severity as the gestational age at birth decreases. Modern neonatal therapies have improved the incidence of pulmonary fibrosis in extremely preterm neonates, yet the issue of alveolar simplification leading to a reduction in the number of alveoli remains a constant concern.

## Personal thanks

I wish to express my gratitude to all the technicians who actively participated in the execution of tissue microarray, as well as those who proficiently executed the procedural technique.
